# Εleven Greek Legume Beans: Assessment of Genotypic Effect on Their Phytochemical Content and Antioxidant Properties

**DOI:** 10.3390/antiox13040459

**Published:** 2024-04-13

**Authors:** Eleni D. Myrtsi, Dimitrios N. Vlachostergios, Christos Petsoulas, Sofia D. Koulocheri, Epameinondas Evergetis, Serkos A. Haroutounian

**Affiliations:** 1 Laboratory of Nutritional Physiology and Feeding, Department of Animal Science, School of Animal Bioscience, Agricultural University of Athens, Iera Odos 75, 11855 Athens, Greece; elenamirtsi@aua.gr (E.D.M.); skoul@aua.gr (S.D.K.); epaev@aua.gr (E.E.); 2 Institute of Industrial and Forage Crops, Hellenic Agricultural Organization ELGO-DIMITRA, 41335 Larissa, Greece; petsoulaschristos@elgo.gr

**Keywords:** legumes, *Fabaceae*, fatty acids, polyphenols, antioxidants, *Cicer arietinum* L., *Pisum sativum* L., *Vicia faba* L., *Lens culinaris* L., *Phaseolus vulgaris* L.

## Abstract

Legumes, one of the first crops of humanity, inherently constitute a staple nutritional source for mankind, attracting significant research attention that has been afforded to the development of numerous cultivars. The study herein concerns the exploitation of the nutritional and bio-functional content of beans harvested from eleven Greek cultivars belonging to five different species, namely *Cicer arietinum* L., *Pisum sativum* L., *Vicia faba* L., *Lens culinaris* L., and *Phaseolus vulgaris* L. The final goal is to define their varietal identity and correlate their phytochemical content with their potential utilization as functional foods and/or feed of high nutritional value. In this respect, their extracts were screened against the presence of 27 fatty acids and 19 phenolic compounds, revealing the presence of 22 and 15 molecules, respectively. Specifically, numerous fatty acids were detected in significant amounts in all but *C. arietinum* extract, while significant polyphenolic content was confirmed only in *P. vulgaris*. Among individual compounds, linoleic acid was the major fatty acid detected in amounts averaging more than 150 mg/g, followed by oleic acid, which was present as a major compound in all extracts. Among the nine polyphenols detected in *P. vulgaris*, the molecules of genistein (3.88 mg/g) and coumestrol (0.82 mg/g) were the most abundant. Their antioxidant properties were evaluated through DPPH and FRAP assays, which were highlighted as most potent in both tests of the *V. faba* extract, while *C. arietinum* was determined as totally inactive, indicating a potential correlation between the phenolic content of the plant species and antioxidant activity. These results are indicative of the significant advances achieved for the cultivars investigated and reveal their important role as nutritional crops for human and animal consumption.

## 1. Introduction

Since antiquity, the Fabaceae family of plants, commonly referred to as legumes, constitute a staple nutrition source for humanity second only to Poaceae (cereals), both marking the transition from foraging to agriculture [1]. Nowadays, grain legumes represent almost 30% of the global grain supply, providing more than one-third of humanity’s dietary proteins [2]. On the other hand, because they display the ability to enhance the growth rate of free-grazing husbandries [3], legume herbal tissues represent one of the primary animal feed sources, either as forage and pasture plants or as raw biomass that is processed in the form of hays or silages [4].

Currently, the cultivation of legumes faces many challenges, comparable to those of cereals, mainly as a sustainable protein source contributing to the global need for food security. These needs are mainly stressed by factors such as climate change [5], population growth, and the shift in consumers’ preferences, which have emerged as central issues for the planning of agriculture and food systems. Nevertheless, the characteristics of *Fabaceae* crops, such as nitrogen fixation and drought resistance [6] favor their expansion in this challenging environment, while their increased content of protein minerals [7], fat, carbohydrates, vitamins, antioxidants [4], and essential amino acids, such as methionine, cystine, and cysteine, advocate their role as a multifunctional sustainable alternative to cereals.

In the near future, it is estimated that the world’s demand for agricultural products will increase by 20%, while the demand for animal-origin protein will grow up to 60% [8]. To cope with this necessity, legume breeding has to re-invent and repeat the vast progress achieved in the mid-20th century for cereals’ yields in order to maintain the legume’s potential for human and animal diets. As a response, plant breeders worldwide develop and produce new varieties, aiming to improve these crops’ environmental adaptability and biotic stress tolerance to increase their yield [9]. Although the aforementioned efforts have provided encouraging results concerning legumes’ role either as meat substitutes in human nutrition or as high protein feeds, their phytochemical content is somehow neglected. The latter is characterized by valuable nutritional elements and numerous bioactive phytochemicals.

In this context, the present study aims to ameliorate the breeding efforts’ results through the exploitation of beans obtained from eleven Greek cultivars that belong to the following five different species: *Cicer arietinum* L., *Pisum sativum* L., *Vicia faba* L., *Lens culinaris* L. and *Phaseolus vulgaris* L. For this purpose, their antioxidant properties were studied along with their content of crucial nutrient elements in the form of fatty acids, anti-nutritional molecules, such as tannins, and bioactive ingredients in the form of polyphenols. This supplementary description of the characteristics of these crops aspires to provide new drivers for plant breeding but also new priorities for the suitability and complementary functions of legumes in human and animal nutrition.

## 2. Materials and Methods

### 2.1. Samples

This study is focused on the Fabaceae family of beans obtained from eleven Greek cultivars, developed by the legume breeding program of the Institute of Industrial and Forage Crops (IIFC) of the Hellenic Agricultural Organization ELGO-DIMITRA. Bean samples were obtained from plants grown in replicated field plots at the central farm of IIFC in Larissa (39°36′ N, 22°25′ E) during the culture period of 2020–2021. When plants reached their maturity levels, their crops were hand-harvested and threshed using a laboratory thresher (Wintersteiger LD350, Ried, Austria) to provide 0.5 kg from each crop that was used for the analyses. Namely, the crops of the following legume species and cultivars have been exploited: *Cicer arietinum* L. [clv. Amorgos (CAA), Gavdos (CAG) and Thiva (CAT)], *Pisum sativum* L. [clv. Olympos (PSO) and Dodoni (PSD)], *Vicia faba* L. [(clv. Polykarpi (VFP) and Tanagra (VFT)], *Lens culinaris* L. [clv. Dimitra (LCD), Thessalia (LCT) and Samos (LCS)], and *Phaseolus vulgaris* L. [clv. Pyrgetos (PVP-1, PVP-2)].

### 2.2. Chemicals and Standards

The analytical grade solvents hexane, dichloromethane, and methanol were obtained from Fisher Chemicals (Hampton, NH, USA) and used for the extractions. The LC–MS/MS determinations were performed using water and acetonitrile purchased from J.T. Baker (Phillipsburg, NJ, USA) and formic acid provided by Fisher Chemicals, all of LC–MS grade quality. Potassium hydroxide (KOH) and boron trifluoride (BF_3_) (14% in methanol) were obtained from Sigma-Aldrich (Burlington, MA, USA), and hydrochloric acid (HCl) was provided by Fluka (Long Island, NY, USA).

All polyphenol standards used for the analyses were purchased from ExtraSythese, except equol and puerarin which were obtained from TCI (Tokyo Chemical Industry, Tokyo, Japan), and calycosin, calycosin-7-*O*-β-d-glucoside, matairesinol, lariciresinol, and secoisolariciresinol, which were purchased from Biosynth Carbosynth (Gardner, MA, USA). The purity of all standards was >95%, expect 3′,4′,7-trihydroxyflavone, the purity of which was >90%. The 2-(4-chlorophenyl) malonaldehyde was provided by Sigma-Aldrich and used as an internal standard. All standards used for the fatty acids’ quantification analysis were included in the Supelco 37 Component FAME Mix from Sigma-Aldrich (St. Louis, MO, USA).

### 2.3. Samples Preparation

Immediately after their collection, beans were placed in a dark and well-ventilated place to dry. An amount of 5 g from each dried bean was powdered and extracted successively with n-hexane, dichloromethane, and methanol according to a method reported by Myrtsi et al. [10]. The extraction solvents were evaporated using a Büchi vacuum pump V-700, Vacuum controller V-850, and Julabo F12 cooling unit in temperatures below 35 °C. The hexane extracts were used for the determination of fatty acids content and the methanolic extracts for the assessment of polyphenols composition [10].

### 2.4. Total Phenolic (TPC) and Tannin (TTC) Contents

TPC and TTC values of the investigated beans were determined by applying previously reported spectrophotometric methods [10] using 96-well microplates (Sarstedt AG and Co. KG, Nümbrecht, Germany) and measuring the respective absorbances on a NanoQuant infinite M200PRO instrument (Tecan Group Ltd., Männedorf, Switzerland). All experiments were performed in triplicate.

Briefly, the TPC was determined by measuring the absorption at a 765 nm wavelength and calculating the corresponding TPC values against a gallic acid standard calibration curve (y = 0.0038258x − 0.0090575, R^2^ = 1). The results are expressed as mg of gallic acid equivalents per g of dry extract (mg GAE/g extract). For the TTC determination, the absorbance was measured at 500 nm wavelength, and the TTC value of each sample was determined using a catechin standard calibration curve (y = (7.261 × 10^−5^)x + 0.048697, R^2^ = 0.9995). The respective results are expressed as mg of catechin equivalents per g of dry extract (mg CE/g extract).

### 2.5. Determination of Polyphenols Composition Using LC–MS/MS Analysis

An Accela ultra-high-performance liquid chromatography system coupled with a TSQ Quantum Access triple quadrupole mass spectrometer (Thermo Fisher Scientific, Inc., Waltham, MA, USA) was used for the determination of the polyphenolic composition of the investigated samples, applying a previously described method by Myrtsi et al. [10]. Specifically, the chromatographic separation of polyphenol analytes was achieved on a C_18_ column (150 mm × 2.1 mm, 3 μm, Fortis Technologies Ltd., Neston, Cheshire, UK) coupled with an AF C18 guard column (10 mm × 2.0 mm, 3 μm, Fortis Technologies Ltd., Neston, Cheshire, UK), and the mobile phase consisted of A, water with 0.1% formic acid, and B, 100% acetonitrile. The flow rate was set at 280 µL/min, and the gradient elution conditions were adjusted as follows: 0.0–2.0 min, 20% B; 2.0–25.0 min, from 20% to 51% B; 25.0–30.0 min, 51% to 70% B; 30.0–30.1 min, 20% B and 30.1–35.0 min, 20% B for the re-equilibration of the column. The injection volume was 10 µL, the sample temperature was set at 25 °C, and the column temperature at 32 °C.

The MS/MS determination was conducted using the electrospray ionization (ESI) technique, and the determination was implemented on the selected reaction monitoring mode (SRM). The capillary temperature was set at 300 °C using nitrogen as a sheath and auxiliary gas supplied by a nitrogen generator (Peak Scientific, Glasgow, UK). The initial gas pressures were set at 35 and 10 Arb, respectively. The collision pressure of the argon gas was adjusted at 1.5 m Torr, and the spray voltage was set at 3.5 kV in both positive and negative polarities. The polyphenols quantification was performed using calibration curves constructed for each analyte and 6 concentration levels using the standard solutions of each analyte and the internal standard solution of a 200 ng/mL concentration. The transitions, collision energies, polarities, retention times (RT), calibration curves equations, and determination coefficients of each analyte are provided as supplementary material (Table S4).

### 2.6. Determination of Fatty Acids Composition Using GC-FID Analysis

A 7820A GC-FID System (Agilent Technologies, Inc., Santa Clara, CA, USA) was used for the determination of fatty acids composition, as has been described previously by Myrtsi et al. [10]. Before each analysis, all fatty acids were esterified. The column used was DB-WAX 30 m, 0.25 mm, 0.25 µm (Agilent Technologies, Inc., Santa Clara, CA, USA). Hydrogen gas (H_2_) was produced from a hydrogen generator (Peak Scientific, Glasgow, UK). Nitrogen (N_2_) as carrier gas and synthetic air, both of high purity, were used. The samples were injected manually (1 µL) in the 10:1 split mode. The oven was programmed as follows: hold at 40 °C for 0.5 min, increase to 195 °C in 25 °C/min increments, then to 205 °C in 3 °C/min and to 230 °C in 8 °C/min increments. The temperature remained at 230 °C for 4 min, then increased to 240 °C for 10 min and to 250 °C for 5 min.

### 2.7. Evaluation of Antioxidant Properties

The evaluation of the antioxidant properties of the investigated samples was implemented using the FRAP (ferric-reducing antioxidant power) and DPPH^•^ (radical scavenging assay) assays, applying a previously described method [10]. All samples were placed in a 96-well microplate in triplicate (Sarstedt AG and Co. KG, Nümbrecht, Germany), and their absorbance was measured with a NanoQuant, infinite M200PRO instrument (Tecan Group Ltd., Männedorf, Switzerland).

For the FRAP assay, the absorbance was measured at a 593 nm wavelength, and the reducing capacity was determined against an FeSO_4_ standard calibration curve (MeOH: y = 0.6213x − 0.19487, R^2^ = 0.9992; Hex, DCM: y= 1.9995x + 0.12137, R^2^ = 0.9994). The respective results are expressed as mmol Fe^2+/^g of extract. For the DPPH^•^ assay, the absorbance was measured at a 515 nm wavelength, and the antioxidant activity was determined against a Trolox calibration standard curve (MeOH: y = 0.3485x + 5.7089, R^2^ = 0.9992; Hex, DCM: y= −0.005353x + 1.0606, R^2^ = 0.9999). The respective results are expressed as mg Trolox Equivalents (TE)/g of dry extract (mg TE/g extract).

### 2.8. Statistical Analysis

The statistical functions of Microsoft Office 365 were used. All results are presented as mean value ± standard deviation (SD) of experiments performed in triplicate. For all calculations performed in this work, the Durbin–Watson (DW) statistical tests for the residuals, the one-way analysis of variance (ANOVA), and the t-test were used. The *p*-value was always less than 0.05.

Hierarchical cluster analysis was utilized to calculate the Euclidean distances between the Fabaceae species and their phytochemical and antioxidant traits, employing the Ward method. Furthermore, a principal component analysis (PCA) was conducted to reduce the dimensionality of the original variables and convert them into orthogonal variables known as principal components. The statistical analyses were carried out using the R-program version 4.3.2 (www.R-project.org, accessed on 2 February 2023).

## 3. Results

The respective yields of the extractions are depicted in Table 1. The hexane extracts of the investigated beans were used for the determination of their fatty acid composition, while their methanolic extracts were used for the measurement of their polyphenolic content. Finally, the assessment of the crops’ antioxidant capacities was performed on all extracts.

### 3.1. Determination of Nutritional Content

#### Fatty Acids Composition

**Figure 1:**
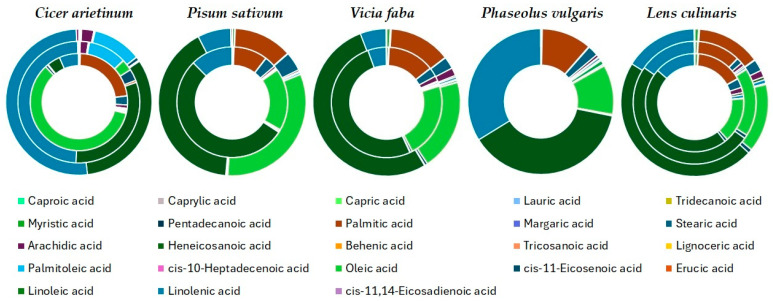
Fatty acids content of the investigated beans.

It is well known that the Fabaceae family of plants exhibits the ability to synthesize a variety of fatty acids. The quantitative presence of the most abundant fatty acids in the studied crops is presented in Figure 1, while the full data of their quantitative determination have been provided as Tables S1–S3 in the Supplementary Materials section.

### 3.2. Polyphenolic Content

#### 3.2.1. Total Phenolic and Tannin Content

The results of total phenolic and tannin content determinations of the methanolic extracts of studied beans are presented in Table 2.

#### 3.2.2. Polyphenolic Composition

The individual phenolic compounds’ fingerprinting was performed by exploiting the presence of the 19 most abundant phenolics in legumes. The respective results of their quantitative presence determination are presented in Table 3, verifying the presence of 11 molecules in the samples studied.

### 3.3. Antioxidant Properties

The antioxidant properties of beans were determined by performing the complementary to each DPPH^•^ and FRAP assays on all the extracts (hexane, dichloromethane, and methanol). The respective experimental results are included in Table 4.

### 3.4. Screening of Fabaceae Family Crops Utilizing Hierarchical Clustering Analysis (HCA) and Principal Component Analysis (PCA)

#### 3.4.1. Principal Component Analysis (PCA)

**Figure 2:**
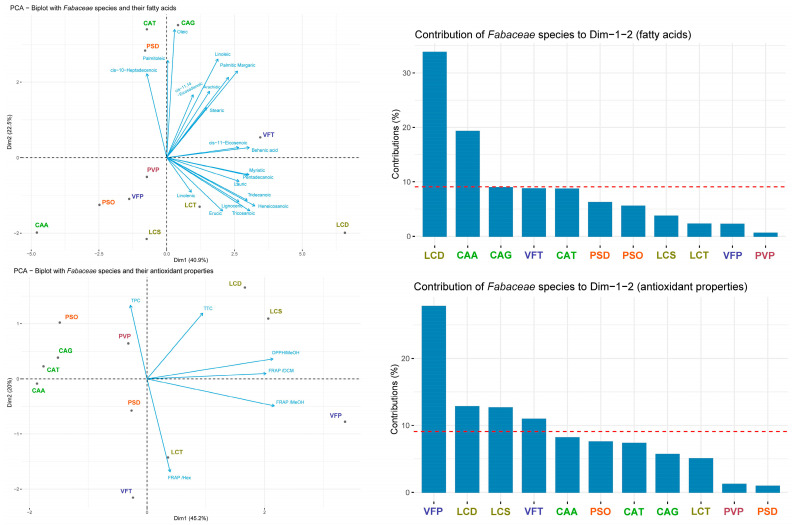
PCA results visualized graphically. (**Above**) The biplot illustrates the relationship between Fabaceae *species* and their fatty acid composition. The arrows represent the fatty acids, while the points represent the Fabaceae species/cultivars. The length and direction of the arrows indicate the correlation between each fatty acid and the principal components. The barplot shows the contribution of each species to the first two principal components. (**Below**) In this biplot, Fabaceae *species* are depicted along with their antioxidant properties. Similarly, the arrows represent different antioxidant properties, while the points represent the Fabaceae species. The length and direction of the arrows indicate the correlation between each antioxidant property and the principal components. The barplot displays the contribution of each species to the first two principal components. The red dashed line represents the average contribution of the species to the first two principal components.

PCA is a statistical method that operates in an unsupervised manner, enabling the detection of underlying patterns within a dataset, thereby unveiling concealed similarities and/or disparities. Specifically, PCA is utilized to unveil correlations between the compositions of phytochemicals and the antioxidant properties across various species, as well as to categorize them. In this respect, two distinct analyses were conducted: one using the dataset comprising fatty acids composition, and the other utilizing the polyphenol content alongside antioxidant properties (Figure 2). In the first PCA, approximately 41% and 22% of the total variation were accounted for by the first (Dim1) and second (Dim2) principal components, respectively, cumulatively explaining 63% of the total variance. Notably, heneicosanoic, palmitic, oleic, and tricosanoic fatty acids exhibited the most substantial contributions to these first two components. As far as the Fabaceae genera, the analysis demonstrated a moderate grouping between them, with two *Cicer arietinum* varieties (CAT and CAG) and three *Lens culinaris* varieties (LCD, LCT, and LCS) being grouped together. Also, LCD, CAA, and CAG display the highest contribution to the first two components.

In the second PCA, the first (Dim1) and second (Dim2) principal components explained 45% and 20% of the overall variation, respectively, thereby collectively elucidating 65% of the total variance. Dim1 exhibited a strong positive correlation with antioxidant capacity, evaluated through FRAP and DPPH assays, as well as with TTC, and a negative correlation with TPC. Dim2 exhibited a marked negative correlation with the FRAP (Hex) assay. This analysis revealed a clearer pattern in the grouping between the genera. However, similar to the first PCA, it is evident that significant variability can also be observed among species within the same genera. Additionally, VFP, LCD, and LCS demonstrate the highest contribution to the first two components.

#### 3.4.2. Hierarchical Cluster Analysis (HCA)

**Figure 3:**
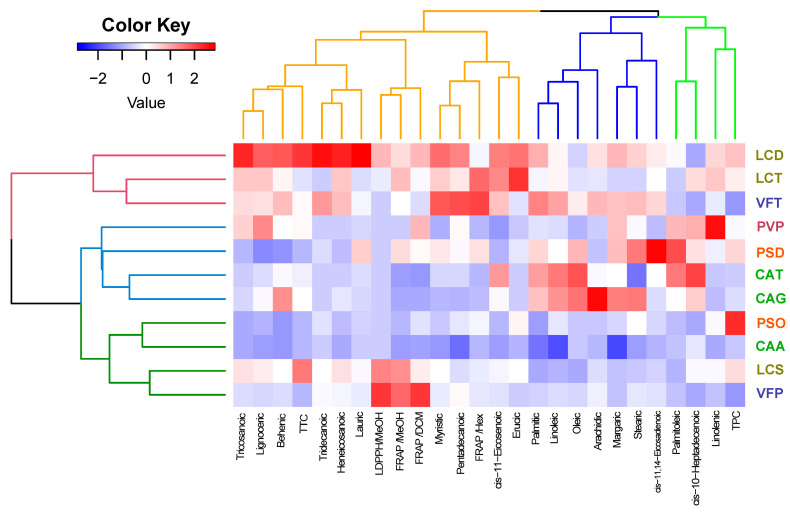
Hierarchical clustering analysis (HCA) was employed to construct a dendrogram, while a heatmap was generated to visualize the distribution of phytochemical properties among species/cultivars within the Fabaceae family.

A classification of the 11 investigated Fabaceae family beans was conducted via HCA, employing the Euclidean distance and the Ward method, while considering 26 traits encompassing phytochemical components and antioxidant properties. According to the HCA results, the cultivars within the Fabaceae family were grouped into three distinct clusters (Figure 3). Cultivars LCD, LCT, and VFT comprised the first group, while PVP, PSD, CAT, and CAG constituted the second group. The final group consisted of PSO, CAA, LCS, and VFP. Additionally, a heatmap was generated to visualize the values of the quality traits. The heatmap further highlights the prominence of the LCD, LCT, and VFT varieties for the investigated traits. Figures depicting the detailed correlation between the content and the varieties are provided as Supplementary Figures S1 and S2.

## 4. Discussion

### 4.1. Nutritional Content

#### 4.1.1. Fatty Acids Composition

It is well established that Fabaceae plants are capable of synthesizing predominantly the acids palmitic, oleic, linoleic, and linolenic among a variety of fatty acids [11]. Except for these four abundant fatty acids, we have revealed herein the presence of palmitoleic acid in high concentrations in the investigated beans. Additionally, in several samples, we also detected the acids stearic and arachidic among the most abundant fatty acids. The benefits from the consumption of dietary long-chain n-3 polyunsaturated fatty acids, such as *α*-linolenic acid, are well documented indicating the necessity of their inclusion in the human diet for normal growth and development [12]. Consequently, the literature abounds with reports concerning the fatty acids content of legume beans, providing a solid background for the assessment of the results obtained herein.

The fatty acids content of *Phaseolus vulgaris* beans has been studied thoroughly, to the extent that they have been utilized as the base substrates for the performance of various pioneering metabolic studies for fatty acids [13]. In addition, the influence of germination was investigated with respect to the content of fatty acids in beans [14], indicating the prevalence of C20–C22 fatty acids in mature beans. This finding is in contradiction with our results, which highlighted the polyunsaturated fatty acids (C18, C21) as the prevailing fatty acids, accounting the 278.9 mg/g of extract. It must be noted however that the literature findings have determined irrelevant the influence of post-harvest treatments on the fluctuation of fatty acid content [15], while other studies investigating the fatty acid content during fruit ripening have reported that the fatty acid content is dominated by saturated components [16]. These reports are in contradiction with our findings indicating that saturated fatty acids comprise only 16.4% of the total fatty acids content of the beans investigated. It must be noted, however, that the more detailed profiling of *P. vulgaris* fatty acid content presents findings that also reveal as major components the acids palmitic, oleic, linoleic, and linolenic [17,18]. These results replicate the findings of Marotii et al. (2020) [19], which exploited the functional features of these widespread legumes on human health and simultaneously underlie the importance of fatty acids profiles as potential markers of “genotype-niche diversity” fingerprints for beans.

**Figure 4:**
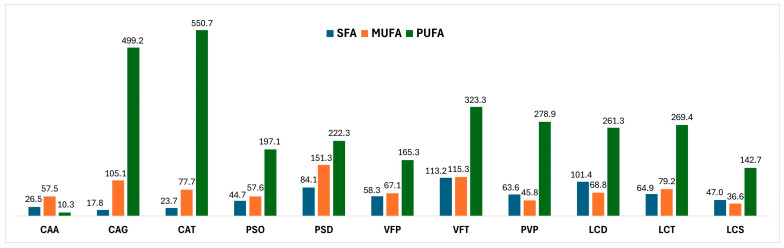
Fatty acids: saturated (SFA), mono-unsaturated (MUFA), and poly-unsaturated (PUFA) content (mg/g extract) of the investigated beans.

Although the fatty acids content of *C. arietinum* has been extensively studied, these studies are not as extensive as for *P. vulgaris*. In an early critical review summarizing the nutritional values of the chickpea [20], fatty acids were not included, while additional preliminary studies soon culminated in a limited profiling of chickpea fatty acids [21]. More recent research findings indicate a prevalence of saturated fatty acids [22]. The latter was not confirmed in our results, which pointed out the CAT and CAG cultivars as particularly rich sources of polyunsaturated fatty acids. A similar fatty acid profile was recently reported on total chickpea fats, where it was determined that 66% consist of polyunsaturated fatty acids, 19% monounsaturated, and only 15% are saturated [23]. These results are in agreement with our findings of CAT and CAG fatty acids profiles, which indicated that, respectively, they comprised 84.45 and 80.25% polyunsaturated fatty acids, 11.92 and 16.89% monounsaturated, and only 3.63 to 2.85% are saturated (Figure 4). Nevertheless, the older reports on the low content of polyunsaturated fatty acids agree with the herein-reported finding for CAA, the third cultivar studied, which was determined to contain significantly lower concentrations of polyunsaturated fatty acids, while monounsaturated fatty acids accounted for 57.52 mg/g of the extract.

The presence of fatty acids in *Pisum sativum* beans has been previously studied with gas chromatography [24,25,26] and was correlated with bean size and agroclimatic conditions [27]. In this context, the acids linoleic, oleic, and linolenic were determined as the most abundant unsaturated fatty acids, with palmitic acid as the prevailing saturated fatty acid. Of these, linoleic acid was mainly detected in small and medium beans, while the presence of palmitic and stearic acid was increased in larger sizes, while the linolenic acid occurs in lower concentrations in all sizes. In the species studied herein, we determined linolenic acid as predominant and palmitic displaying the lowest concentration in PSO cultivars, a finding presumably related to the size of the bean. A literature study on pea lipids using LC/ESI-MS/MS has confirmed this distribution of fatty acids and showed that they are mainly composed of polyunsaturated fatty acids, ranging from 42.01% to 60.68% of the total fatty acids, with a relatively low content of unsaturated fatty acids (17.46–24.95%) [28]. The herein-studied PSO and PSD species approach the above literature data, as they are composed, respectively, of 65.84% and 48.57% of polyunsaturated fatty acids. Only PSD shows higher percentages in unsaturated fatty acids since their amount was increased probably because of their beans’ size.

The first studies for the determination of *Vicia faba* beans’ fatty acids were implemented by Duc et al. (1999) [29], reporting the presence of linoleic acid as predominant, which was followed by the acids oleic acid and palmitic. In contrast to these results, a study by Akpinar et al. (2001) [30] showed that the major unsaturated fatty acid in *Vicia faba* seeds was oleic, followed by linoleic and linolenic acids, while palmitic and stearic acids were the major saturated fatty acid components [30]. This discrepancy in results may be rationalized by considering that these studies concerned different samples. Nevertheless, Duc’s results were recently confirmed by a newer report [31] and our work, since we detected linoleic acid at a higher percentage while observing that a high level of unsaturated fatty acids enhances the nutritional value of *Vicia faba* beans.

The fatty acid content of *Lens culinaris* is well studied for several types of lentils, which have determined linoleic as the main fatty acid and a 4:1 ratio of unsaturated to saturated fatty acids. In addition, these studies indicated that lentil oil contains 44% linoleic and 12% linolenic acid, with their oil content being 1.5–3.0% [32]. More recent studies on many lentil varieties confirmed the predominance of linoleic acid, followed by oleic, palmitic, and linolenic acids [33]. Comparable results with those in the literature were obtained herein concerning the polyunsaturated fatty acids content and indicating that lentil extracts contain mainly unsaturated fatty acids (80.93–84.38%), including monounsaturated fatty acids (17.08–30.58%) and polyunsaturated fatty acids (52.24−63.85%) [34].

#### 4.1.2. Tannin Composition

With respect to the TTC values οf the investigated samples, the beans of lentil cultivars were determined to be the most abundant in tannins, displaying values ranging from 124.1 to 95.4 and 20.2 mg CE/g for the LCD, LCS, and LCT extracts, respectively. Chickpea cultivars exhibited mixed results with two extracts containing moderate tannin concentrations, which ranged from 38.8 to 25.3 mg CE/g for the CAG and CAT extracts, respectively, while tannins were not detected in the third cultivar (CAA), the two pea cultivars (PSO and PSD), and one fava bean cultivar (VFP). In the two remaining extracts, the tannin content ranged from 38.5 to 38.3 mg CE/g in the kidney (PVP) and fava (VFT) bean extracts respectively. These findings indicate that tannins’ content is strongly dependent upon the variety. For example, among lentil cultivar beans, LCT was determined to display a TTC value five times lower compared to LCD and LCS, advocating the aforementioned finding.

The previous literature findings have documented the rich tannin content of kidney beans [35], with values ranging from 0.03 to 1.56 mg CE/g extract [36]. These findings were expanded herein since the detected concentration levels ranged from 0.27 to 36.87 mg CE/g extract. These values are in counter analogy with the storage time and process [37], advocating the placement of the present results within the range of already detected tannin contents. On the other hand, the tannin content of *Pisum sativum* beans was significantly low but within the wide range of the literature-reported values [38]. The content of concentrated tannins in peas is mainly genotype dependent [39], which enhances the use of the crops studied in the diet because of the low levels of these anti-nutritional compounds. The total tannin content (TTC) in various faba bean cultivars differed significantly since the “Tanagra” cultivar (VFT) displayed a moderate tannin content (1.7 g/kg), which is in line with the literature (0.06 to 14.5 g/kg) [40]. On the other hand, the methanolic extract of VFP beans was determined to display a poor tannin and phenolic content, in contrast with the literature reports revealing lentil crops as a rich source of tannins [41].

### 4.2. Polyphenolic Composition

Among all methanolic extracts studied, the highest TPC value was determined for the extract of *Pisum sativum* (PSO) beans (26.2 mg GAE/g extract), a value almost double compared to the methanolic extract of *Lens culinaris* beans (LCD) which displayed the second highest value (14.5 mg GAE/g extract). The presented herein values range from 26.2 mg GAE/g for PSO extract to a lower detection limit for VFP and VFT extracts. Specifically, the two pea cultivars uniformly display an increased phenolic content, ranging from the upper limit (PSO) to 12.7 mg GAE/g for PSD extract, followed by the three lentil cultivars ranging from 14.5 to 12.3 and 10.5 mg GAE/g for the LCD, LCS, and LCT extracts, respectively. In between lentils and chickpeas lies the kidney bean (PVP) with 8.2 mg GAE/g of extract. Finally, the chickpea cultivars’ phenolic content ranges from 5.8 to 4.4 and 4.2 mg GAE/g for the CAG, CAT, and CAA extracts, respectively. The total phenolic content is widely studied in numerous research studies concerning legume beans, most often coupled with antioxidant bioassays. This vast availability of TPC results for numerous Fabaceae *taxa* enables a thorough review of our results for each *taxon*.

It is noticeable, however, that this is not the case for phenolic fingerprinting, since the respective studies are scarce for *Fabaceae* plants. Research endeavors concerning the determination of the individual phenols’ presence and concentrations in legume crops mainly concern the polyphenolic content of a limited number of beans and only specific metabolites. For example, there are several studies on the polyphenolic content of *C. arietinum* beans, which are commonly known as chickpeas and used widely in human diets. These studies concern only specific metabolites, such as the isoflavones biochanin A, formononetin, genistein, calycosin, ononin, and sissotrin [42]. Of these six compounds, only ononin was determined herein in CAA cultivar beans. Therefore, discussion on this subject is not always possible since the extant phenolic fingerprinting of their extracts comprises an endeavor presented herein for the first time.

The phenolic content of kidney beans (*P. vulgaris*) has been extensively studied around the world, for cultivars originating from Africa [36], America [43], Asia [44], and Europe [45]. Elements of variance between these reports highlight one of the biggest problems concerning the research results that exploit different methodologies for the same subject. Specifically, in these four studies, the total phenolic content is expressed per extract in equivalents of gallic acid [35] and catechin [36,37], along with their absolute concentration per dry matter [34]. Thus, only the African varieties’ results are presented in a comparable mode with our results, revealing a total phenolic concentration ranging from 4.4 to 2.34 mg GAE/g of extract [36]. This indicated the prevalence of the PVP cultivar in relation to its TPC value, a finding also confirmed by more recent studies indicating that its TPC value ranges from 1.3 to 0.3 mg GAE/g of extract for the investigated 33 cultivars [46]. On the other hand, although scarcer, the phenolic fingerprinting of kidney beans is in line with the findings herein that confirm the dominance of flavonoids found in previous studies [47,48]. Finally, with respect to the individual molecular structures, our results expand to a greater range of the presence of phenolic compounds, revealing the detection—for the first time—of nine phenolic compounds comprising kidney beans’ ingredients.

Previous studies on lentil beans’ phenolic content have focused on their TPC detection [49,50], reporting values that confront with herein-presented measurements, displaying values ranging from 10.5 to 12.3 and 14.5 mg GAE/g for the methanolic extracts of LCT, LCS, and LCD, respectively. It is evident that these values differ from those determined in previous studies for extracts utilizing acetone–water extraction [51,52]. On the other hand, a common finding of most previous studies concerning the diversification of phenolic content within cultivars was also confirmed by the present results. An extensive investigation of lentil bean coats has determined as major phenolic components the acids gallic, protocatechuic, and p-coumaric, along with the molecules (+)-catechin and (–)-epicatechin and the glycol flavones luteolin-7-glucoside, apigenin-7-glucoside, and apigenin 7-apioglucoside. The molecule of luteolin was detected as a free flavone, along with various procyanidins [53]. This husk profile was also verified for their beans [51], confirming the generic profile of herein-investigated cultivars, especially with respect to the procyanidins content.

Phenols comprise one of the most important bioactive compounds in peas. Previous studies showed that the total phenolic content depends on the genotype, color variation, and shape of the seed hull, with green or orange seed hulls showing the highest TPC values [54]. These results have provided a large variation among measurements, justifying the observed-in-our-study variance between PSO and PSD cultivars, which displayed the highest concentrations of total phenolics (respectively, 26.2 and 12.7 mg GAE/g extract) that are within the range of the literature measurements [55]. Although polyphenols have been found in peas both as free and conjugated forms, in the present study, we mainly detected their glycosides in our methanolic extracts, presumably because most of the free polyphenols were extracted by dichloromethane. Thus, from flavonols, the molecules of quercitrin [56] and isoquercetin [38,57] were detected in pea hull. Additionally, procyanidins B1 and B2 were detected in the phenolic-richest sample, comprising the first report of procyanidin B1 presence in pea beans [41]. With respect to the presence of phytoestrogens, the presence of their precursor isoliquiritigenin was detected in both samples, a finding in line with the literature [55,58]. It is also noticeable that trace amounts of the phytoestrogen glycosides glycitin [55,58] and ononin [55] were detected, along with chlorogenic acid, which was the most frequently detected phenolic acid in pea-pod extract, and was found only in the pea cultivar with the lowest phenolic content [58,59]. Finally, the presence of quercetagetin-7-O-glucoside in their pods is reported herein for the first time.

The TPC value of the methanolic extract of fava beans was not determined, as it was below the detection limit. This result is in contrast with the literature data reporting low TPC values in fava beans compared to fava leaves [40,60,61,62,63]. This result is probably related to the removal of the bioactive components during the sequential extraction [33,34,35], while the possible variation of TCP and phenolic composition among cultivars is related to the different maturity stages of beans and the accuracy of the method [62]. Herein, the detection of phenolic compounds in traces is in accordance with the limited content of total phenolics. More specifically, among compounds reported in the literature, quercitrin a *Vicia faba*-peel polyphenol, was detected in both samples [64], while epigallocatechin gallate was detected only in the VFT cultivar [65].

### 4.3. Antioxidant Properties

The results of antioxidant capacity determination of the studied extracts differ greatly between the two methodologies applied. The free-radical scavenging bioassay (DPPH^•^) provided significant results for methanolic extracts of the investigated three chickpea cultivars, with values fluctuating from 5 to 3.5 and 0.9 mg TE/g for LCD, LCT, and LCS extracts, respectively. Hexane extract of the PSD pea cultivar was also enumerated to 3.3 TE/g extract, and the VFP fava bean cultivar exhibited antioxidant potency for both hexane and methanolic extract, with values between 0.7 and 7 TE/g extract. For the remaining extracts, the respective results were beyond the methodological level of detection. These results may be traced to another peculiarity of methanolic extracts, reporting that LCD, LCT, and LCS extracts contain anthocyanidins as common compounds. The activity of fava bean VFP cultivar methanolic extract cannot be attributed to its phenolic content, which is very poor and has to be further investigated in future research. Similarly, the hexane extracts of PSD and VFP present an analogous fatty acid profile, which may be considered for the explanation of the DPPH^•^ results.

With respect to the reducing ability bioassay (FRAP) findings, they presented a uniformly positive result throughout the range of the extracts. Specifically, hexane extracts displayed moderate activity, ranging from 0.08 to 0.46 TE/g extract, dichloromethane extracts were determined as the most potent ranging from 0.23 to 1.77 TE/g extract, and methanolic extracts of weaker activity ranging from <0.01 to 0.23 TE/g extract. With respect to their phytochemical content, each extract’s fatty acid content may provide a viable substrate for the determination of structure–activity relationships. In the same manner, the lower activity of methanolic extracts can be attributed to their relatively low phenolic content. On the other hand, the dichloromethane extract activities cannot be explained with the present results and are already under further phytochemical investigation, which is indicated as primary among numerous classes of ingredients, including phenols, flavonoids, and carotenoids.

Specifically, kidney beans have been previously found to exhibit low free-radical scavenging activity [37,66], while their reduction activity (FRAP) was compatible with previous reports [67]. The three chickpeas’ cultivars studied herein reveal a moderate-to-low antioxidant capacity in the reduction bioassay, ranging from below 0.1 to 0.32 mmol Fe(II)-E/g extract. The respective results for chickpea bean extracts highlighted yellow peas as the most active [68], affirming our results. In addition, other studies also confirm the herein-reported FRAP results [69] providing a potential explanation for DPPH inactivity results, attributing this phenomenon to the fact that DPPH^∙^ reactions depend greatly on the presence of dissolved oxygen in the reaction mixture, resulting in relatively low antioxidant activity [70].

Finally, the measurements for the three lentil cultivars are in line with previous reports on FRAP bioassays [71] although a significant diversification was observed with the previous DPPH results. Specifically, our results fluctuated from 5 to 3.5 and 0.9 mg TE/g extract for LCS, LCD, and LCT [72], while the connection between TPC and antioxidant activity determined in other studies [49] was not replicated herein, suggesting a relevance between the procyanidin content and the antioxidant activity. These results are in line with the literature reports for *V. faba,* which have demonstrated that immature faba crops exhibit better antioxidant activities compared to mature crops [73].

### 4.4. Fabaceae Family Screening Utilizing Hierarchical Clustering Analysis (HCA) and Principal Component Analysis (PCA)

Hierarchical clustering analysis (HCA) and principal component analysis (PCA) are both potent statistical methodologies used to elucidate patterns in the relationships between various genetic materials based on their phytochemical traits [22]. Combining the fatty acid composition, polyphenol content, and antioxidant assays resulted in a classification where cultivars LCD, LCT, and VFT emerged as the most highly valued varieties. Both PCAs produced a moderate grouping between genera and only for *Lens culinaris* and *Cicer arietinum*. These findings underscore how cultivars influence quality and emphasize the potential utility of these traits as markers in *Fabaceae* breeding programs.

## 5. Conclusions

The studied herein legume beans play an important role in both human and livestock nutrition, especially in periods of food scarcity. These plants also comprise a rich source of valuable metabolites with many beneficial effects on consumers’ health. The present study concerns the exploitation of legume-cultivar beans, highlighting them as a rich source of nutrients and precious metabolites. The final goal is to exploit their phytochemical content in correlation with their varietal identity, aiming to determine their utility as functional foods and/or high-nutritional value animal feed. It must be noted that, in combination with their high protein content, legume consumption can offer a plethora of health benefits.

With respect to the effect of the cultivars on the phytochemical and antioxidant profile of the legumes studied, it was revealed that there is a significant variation among cultivars. This finding highlights the concept of the “varietal identity” for legume-based products, especially those that can be used as functional foods. Characteristic examples of this variation are the difference in the fatty acid content between chickpea cultivars or the multiple differences among lentil cultivars with respect to their total tannin content. Undoubtedly, further multidisciplinary research is needed to fully unveil the effect of genotype and other factors influencing their characteristics.

## Figures and Tables

**Table 1:** Extraction efficiency (as % yield) for hexane (Hex), dichloromenthane (DCM), and methanol (MeOH) solvents.

Abbreviation	Samples	Extraction Yield (%)		
		Hex	DCM	MeOH
CAA	*Cicer arietinum* (clv Amorgos)	3.15	1.46	5.89
CAG	*Cicer arietinum* (clv Gavdos)	3.40	1.65	9.12
CAT	*Cicer arietinum* (clv Thiva)	2.97	1.99	5.88
PSO	*Pisum sativum* (clv Olympos)	0.79	0.86	4.39
PSD	*Pisum sativum* (clv Dodoni)	1.25	0.71	4.48
VFP	*Vicia faba* (clv Polykarpis)	0.66	0.47	0.49
VFT	*Vicia faba* (clv Tanagra)	0.66	0.53	4.43
PVP	*Phaseolus vulgaris* (clv Pyrgetos)	1.06	0.73	4.23
LCD	*Lens culinaris* (clv Dimitra)	0.29	0.75	3.58
LCT	*Lens culinaris* (clv Thessalia)	1.16	0.92	5.36
LCS	*Lens culinaris* (clv Samos)	0.31	1.17	4.50

**Table 2:** Total Tannin (TTC) and Phenolic (TPC) content of Fabaceae beans.

Samples	TPC (mg GAE/g Extract)	TTC (mg CE/g Extract)
*Cicer arietinum* (clv Amorgos)	4.2 ± 0.1 *	<LOD
*Cicer arietinum* (clv Gavdos)	5.8 ± 0.1 *	35.8 ± 20.9 *
*Cicer arietinum* (clv Thiva)	4.4 ± 0.0 *	25.3 ± 15.5 **
*Pisum sativum* (clv Olympos)	26.2 ± 6.0 ***	<LOD
*Pisum sativum* (clv Dodoni)	12.7 ± 0.3 *	<LOD
*Vicia faba* (clv Polykarpis)	<LOD	<LOD
*Vicia faba* (clv Tanagra)	<LOD	38.3 ± 22.4 *
*Phaseolus vulgaris* (clv Pyrgetos)	8.2 ± 0.2 *	38.5 ± 32.4 *
*Lens culinaris* (clv Dimitra)	14.5 ± 0.5 *	124.1 ± 76.6 *
*Lens culinaris* (clv Thessalia)	10.5 ± 0.8 ***	20.2 ± 12.2 *
*Lens culinaris* (clv Samos)	12.3 ± 0.9 *	95.4 ± 56.6 *

GAE: gallic acid equivalent; CE: catechin; <LOD: below the limit of detection; *p*-value correlating samples with the corresponding standard solution: * *p* ≤ 0.005, ** *p* ≤ 0.01, *** *p* ≤ 0.03.

**Table 3:** Polyphenolic composition of Fabaceae beans studied (mg/g extract).

Compound	CAA	CAG	CAT	PSO	PSD	VFP	VFT	PVP	LCD	LCT	LCS
Apigenin	-	-	-	-	-	-	-	0.035 ± 0.000 ***	-	-	-
Calycosin	-	-	-	-	-	-	-	-	-	-	0.006 ± 0.000 *
Chlorogenic acid	-	-	-	-	tr	-	-	-	tr	-	-
Coumestrol	-	-	-	-	-	-	-	0.823 ± 0.150 *	-	-	-
Daidzein	-	-	-	-	-	-	-	0.293 ± 0.000 ***	-	-	-
Daidzin	-	-	-	-	-	-	-	0.075 ± 0.006 ***	-	-	-
Diosmetin	-	-	tr	-	-	-	-	tr	-	-	-
Epigallocatechin gallate	-	tr	tr	-	-	-	tr	-	-	-	-
Genistein	-	-	-	-	-	-	-	3.880 ± 0.004 ***	-	-	-
Glycitein	-	-	-	-	-	-	-	0.011 ± 0.000 *	-	-	-
Glycitin	-	tr	-	tr	-	-	-	-	-	-	tr
Hydroxytyrosol	-	-	-	-	-	-	-	0.258 ± 0.018 ****	-	-	-
Isoliquiritigenin	-	-	-	tr	tr	-	-	-	-	-	0.002 ± 0.000 **
Isoquercetin	tr	tr	tr	tr	tr	tr	tr	tr	tr	tr	tr
Ononin	tr	tr	-	tr	tr	tr	tr	-	-	tr	-
Procyanidin B1	-	-	-	tr	-	-	-	-	0.001 ± 0.000 *	-	0.002 ± 0.001 *
Procyanidin B2	-	-	-	tr	-	-	-	-	0.015 ± 0.002 *	0.006 ± 0.001 *	0.032 ± 0.005 *
Quercetagetin-7-*O*-glucoside	-	-	-	tr	-	tr	tr	-	tr	-	-
Quercitrin	tr	-	-	tr	tr	tr	tr	-	tr	tr	tr

tr: <0.001 mg/g; nd: not detected; *p*-value correlating samples with corresponding standard solution: * *p* ≤ 0.005, ** *p* ≤ 0.01, *** *p* ≤ 0.03, **** *p* ≤ 0.05.

**Table 4:** Antioxidant assay results, DPPH and FRAP, in Fabaceae bean extracts.

Sample	DPPH (mg TE/g Extract)			FRAP (mmol Fe(II)-E/g Extract)		
	Hex	DCM	MeOH	Hex	DCM	MeOH
*Cicer arietinum* (clv Amorgos)	<LOD	<LOD	<LOD	0.08 ± 0.00 *	0.27 ± 0.01 *	<0.01 *
*Cicer arietinum* (clv Gavdos)	<LOD	<LOD	<LOD	0.09 ± 0.00 ***	0.32 ± 0.00 *	0.02 ± 0.00 *
*Cicer arietinum* (clv Thiva)	<LOD	<LOD	<LOD	0.08 ± 0.00 **	0.23 ± 0.01 *	<0.01 *
*Pisum sativum* (clv Olympos)	<LOD	<LOD	<LOD	0.17 ± 0.00 *	0.59 ± 0.00 *	0.03 ± 0.00 *
*Pisum sativum* (clv Dodoni)	3.3 ± 0.2 *	<LOD	<LOD	0.24 ± 0.00 *	0.81 ± 0.01	0.13 ± 0.01 ***
*Vicia faba* (clv Polykarpis)	0.7 ± 0.3 *	<LOD	7.0 ± 1.0 **	0.15 ± 0.00 *	1.77 ± 0.02 *	0.23 ± 0.01 *
*Vicia faba* (clv Tanagra)	<LOD	<LOD	<LOD	0.46 ± 0.01 *	0.56 ± 0.00 *	0.10 ± 0.00 *
*Phaseolus vulgaris* (clv Pyrgetos)	<LOD	<LOD	<LOD	0.08 ± 0.00 *	1.13 ± 0.00 *	0.05 ± 0.00 *
*Lens culinaris* (clv Dimitra)	<LOD	<LOD	3.5 ± 1.7 *	0.17 ± 0.01 *	1.13 ± 0.02 *	0.13 ± 0.00 **
*Lens culinaris* (clv Thessalia)	<LOD	<LOD	0.9 ± 0.4 *	0.41 ± 0.02 *	0.72 ± 0.01 *	0.16 ± 0.01 **
*Lens culinaris* (clv Samos)	<LOD	<LOD	5.0 ± 0.4 **	0.16 ± 0.00 *	0.87 ± 0.02 *	0.20 ± 0.00 *

TE: Trolox equivalent; Fe(II)-E: Fe(II) equivalent; <LOD: below the limit of detection; *p*-value correlating samples with the corresponding standard solution: * *p* ≤ 0.005, ** *p* ≤ 0.01, *** *p* ≤ 0.03.

## Data Availability

The data are contained within the article.

## Supplementary Materials

The following supporting information can be downloaded at: https://www.mdpi.com/article/10.3390/antiox13040459/s1, Table S1: Retention time (RT), calibration curve equation, and determination coefficient (R2) of each fatty acid; Table S2: Fatty acids composition (mg/g extract) of investigated cultivars: Cicer arietinum and Pisum sativum; Table S3: Fatty acids composition (mg/g extract) of investigated cultivars: Vicia faba, Phaseolus vulgaris, and Lens culinaris; Table S4: Transition, collision energy, polarity, retention time (RT), calibration-curve equation, and determination coefficient of each analyte; Figure S1: Matrix of the Pearson correlation coefficients; Figure S2: Correlation heatmap depicting the relationships between phytochemical components and antioxidant properties.
