# Supplementary material for: Εleven Greek Legume Beans: Assessment of Genotypic Effect on Their Phytochemical Content and Antioxidant Properties
Source: Antioxidants (Basel). 2024 Apr 13;13(4):459. doi: 10.3390/antiox13040459 (PMC11047335; doi:10.3390/antiox13040459)
Supplement: Supplementary file 1 [file antioxidants-13-00459-s001.zip › antioxidants-2924935-supplementary.pdf]

Eleven Greek legume beans: Assessment of genotypic effect on their phytochemical content and antioxidant properties

Eleni D. Myrtsi, Dimitrios N. Vlachostergios, Christos Petsoulas, Sofia D. Koulocheri, Epameinondas Evergetis and Serkos A. Haroutounian*

Supplementary Material

Table S1. Retention time (RT), calibration curve equation and determination coefficient (R^2) of each fatty acid.

Table S2. Fatty acids composition (mg/g extract) of investigated cultivars: *Cicer arietinum* and *Pisum sativum*.

Table S3. Fatty acids composition (mg/g extract) of investigated cultivars: *Vicia faba, Phaseolus vulgaris and Lens culinaris*.

Table S4. Transition, collision energy, polarity, retention time (RT), calibration curve equation and determination coefficient of each analyte.

Figure S1: Matrix of the Pearson correlation coefficients.

Figure S2 Correlation heatmap depicting the relationships between phytochemical components and antioxidant properties.

Table S1. Retention time (RT), calibration curve equation, and determination coefficient (R^2) of each fatty acid.

Fatty Acid	RT (min)	Equation	R^2
Butyric Acid	4.10	y=5.43776+1.81190x	0.9990
Caproic acid	5.21	y=–0.32585+2.19335x	0.9990
Caprylic acid	6.33	y=1.83230+2.45992x	0.9992
Capric acid	7.40	y=4.62271+2.62696x	0.9990
Undecanoic acid	7.95	y=2.18465+2.72111x	0.9990
Lauric acid	8.56	y=3.92115+2.76115x	0.9994
Tridecanoic acid	9.23	y=3.78551+2.70616x	0.9992
Myristic acid	10.04	y=11.43893+2.62566x	0.9991
Myristoleic acid	10.52	y=5.60554+2.62292x	0.9991
Pentadecanoic acid	10.95	y=6.88948+2.58981x	0.9990
cis-10-Pentadecenoic acid	11.64	y=6.53148+2.60193x	0.9990
Palmitic acid	11.98	y=17.49106+2.59396x	0.9992
Palmitoleic acid	12.26	y=6.01822+2.57637x	0.9992
Margaric acid	12.97	y=5.61680+2.52989x	0.9990
cis-10-Heptadecenoic acid	13.26	y=5.60661+2.58709x	0.9990
Stearic acid	14.10	y=11.61671+2.58183x	0.9993
Oleic acid	14.39	y=16.92763+2.57653x	0.9992
Linoleic acid	15.05	y=6.21919+2.50695x	0.9993
Linolenic acid	15.49	y=3.36202+2.43641x	0.9994
Linolelaidic acid	16.01	y=3.05237+2.44261x	0.9993
Arachidic acid	17.01	y=5.38288+2.61615x	0.9994
cis-11-Eicosenoic acid	17.44	y=3.58500+2.57952x	0.9995
cis-11,14-Eicosadienoic acid	18.34	y=1.96961+2.50940x	0.9990
Heneicosanoic acid	18.84	y=–8.40315+1.84368x	0.9991
cis-11,14,17-Eicosatrienoic acid	19.28	y=–11.10622+1.42617x	0.9983
cis-5,8,11,14,17-Eicosapentaenoic acid	19.59	y=4.48371+2.38905x	0.9991
Arachidonic acid	20.75	y=4.24675+2.28699x	0.9991
Behenic acid	20.77	y=8.15731+3.70587x	0.9990
Erucic acid	21.27	y=4.69676+2.52971x	0.9991
cis-13,16-Docosadienoic aci	22.37	y=6.27324+2.41700x	0.9994
Tricosanoic acid	23.09	y=1.88253+2.54624x	0.9997
Lignoceric acid	25.99	y=3.94197+2.51159x	0.9991
cis-4,7,10,13,16,19-Docosahexaenoic acid	26.77	y=–8.77566+3.26195x	0.9982
Nevronic Acid	26.88	y=–9.56931+1.30621x	0.9982

Table S2. Fatty acids composition (mg/g extract) of investigated cultivars: *Cicer arietinum* and *Pisum sativum*.

**Compounds**	**CAA**	**CAG**	**CAT**	**PSO**	**PSD**
Saturated Fatty Acid (SFA)					
Caproic acid	nd	nd	nd	nd	nd
Caprylic acid	nd	nd	nd	nd	0.044±0.004
Capric acid	nd	nd	nd	nd	0.002±0.001
Lauric acid	nd	0.024±0.002	0.030±0.005	0.016±0.001	0.182±0.014
Tridecanoic acid	nd	nd	nd	0.009±0.000	0.007±0.002
Myristic acid	0.376±0.004	0.785±0.044	1.260±0.102	1.040±0.021	2.223±0.137
Pentadecanoic acid	0.093±0.000	nd	nd	0.484±0.000	0.538±0.032
Palmitic acid	21.129±0.023	0.307±0.015	0.423±0.023	29.438±0.587	58.827±0.798
Margaric acid	0.075±0.006	0.830±0.042	1.665±0.015	0.321±0.015	0.524±0.024
Stearic acid	2.812±0.056	0.316±0.014	0.603±0.033	9.759±0.743	18.991±0.738
Arachidic acid	1.062±0.018	13.305±0.613	17.692±1.763	2.000±0.219	1.765±0.124
Heneicosanoic acid	0.200±0.000	0.320±0.022	0.474±0.079	0.420±0.035	0.394±0.024
Behenic acid	0.33±0.004	0.844±0.046	0.595±0.091	0.351±0.062	0.281±0.032
Tricosanoic acid	0.184±0.097	0.471±0.031	0.490±0.099	0.210±0.039	0.314±0.033
Lignoceric acid	0.281±0.004	0.558±0.040	0.459±0.081	0.632±0.102	nd
Mono Unsaturated Fatty Acid (MUFA)					
Palmitoleic acid	0.431±0.061	62.965±2.285	70.364±0.108	0.396±0.006	1.915±0.088
cis-10-Heptadecenoic acid	0.131±0.001	0.635±0.029	0.404±0.028	nd	0.265±0.013
Oleic acid	55.92±0.879	18.757±1.960	nd	54.741±2.969	147.208±6.757
cis-11-Eicosenoic acid	0.812±0.073	19.137±1.101	5.114±0.967	1.221±0.145	1.161±0.074
Erucic acid	0.222±0.007	3.591±0.247	1.816±0.356	1.205±0.16	0.794±0.078
Poly Unsaturated Fatty Acid (PUFA)					
Linoleic acid	4.115±0.382	193.066±5.937	212.420±6.532	159.934±8.782	187.511±8.856
Linolenic acid	6.019±0.088	304.018±13.827	334.494±15.213	36.869±2.158	33.243±1.596
cis-11,14-Eicosadienoic acid	0.119±0.002	2.139±0.120	3.795±0.681	0.276±0.032	1.563±0.136
SFAs (% of total Fatty Acids)	28.14	2.85	3.63	14.93	18.37
MUFAs (% of total Fatty Acids)	60.99	16.89	11.92	19.23	33.06
PUFAs ((% of total Fatty Acids)	10.87	80.25	84.45	65.84	48.57

[1] nd: not detected.

Table S3. Fatty acids composition (mg/g extract) of investigated cultivars: *Vicia faba, Phaseolus vulgaris and Lens culinaris*.

**Compounds**	**VFP**	**VFT**	**PVP**	**LCD**	**LCT**	**LCS**
Saturated Fatty Acid (SFA)						
Caproic acid	nd	nd	nd	nd	0.685±0.004	nd
Caprylic acid	nd	nd	nd	nd	nd	nd
Capric acid	nd	nd	nd	nd	nd	nd
Lauric acid	0.052±0.013	0.073±0.007	0.020±0.002	0.517±0.005	0.030±0,000	0.078±0.008
Tridecanoic acid	0.019±0.008	0.069±0.008	nd	0.126±0.002	nd	0.025±0.004
Myristic acid	1.518±0.200	4.207±0.198	0.625±0.028	3.911±0.004	2.613±0.022	1.866±0.160
Pentadecanoic acid	0.591±0.07	1.156±0.066	0.601±0.022	0.98±0.017	0.664±0.003	0.443±0.047
Palmitic acid	39.678±4.248	74.868±3.675	43.105±1.299	67.187±0.066	46.549±0.440	31.751±3.111
Margaric acid	0.289±0.044	0.522±0.028	0.521±0.014	0.536±0.001	0.315±0.003	0.285±0.034
Stearic acid	7.679±1.147	14.513±0.444	9.079±0.191	12.858±0.110	6.309±0.055	5.778±0.590
Arachidic acid	4.424±0.607	9.633±0.118	2.409±0.021	7.456±0.027	3.804±0.052	3.397±0.329
Heneicosanoic acid	0.986±0.115	1.869±0,000	0.586±0.000	3.462±0.015	1.771±0.021	1.493±0.115
Behenic acid	1.343±0.214	2.926±0.061	1.931±0.025	4.347±0.047	2.111±0.030	1.854±0.131
Tricosanoic acid	0.571±0.096	1.165±0.039	1.258±0.015	nd	nd	nd
Lignoceric acid	1.103±0.181	2.24±0.133	3.475±0.139	nd	nd	nd
Mono Unsaturated Fatty Acid (MUFA)						
Palmitoleic acid	0.435±0.056	0.473±0.025	1.269±0.062	0.785±0.004	0.503±0.010	0.333±0.046
cis-10-Heptadecenoic acid	nd	nd	0.379±0.01	nd	0.284±0.002	0.174±0.024
Oleic acid	64.416±8.733	110.877±4.155	42.973±0.22	63.927±0.367	74.496±0.146	34.063±3.508
cis-11-Eicosenoic acid	1.762±0.45	3.189±0.050	0.746±0.007	4.134±0.009	3.957±0.130	2.062±0.212
Erucic acid	0.492±0.077	0.736±0.006	0.468±0.001	nd	nd	nd
Poly Unsaturated Fatty Acid (PUFA)						
Linoleic acid	149.372±18.478	291.238±10.950	147.758±2.988	202.756±1.318	206.005±1.694	106.097±10.734
Linolenic acid	15.621±1.969	31.364±1.146	130.988±6.073	57.981±0.478	62.856±0.515	36.308±3.669
cis-11,14-Eicosadienoic acid	0.341±0.05	0.670±0.024	0.168±0.002	0.587±0.023	0.491±0.121	0.261±0.032
SFAs (%** of total Fatty Acids**)	20.04	20.52	16.38	23.49	15.69	20.76
MUFAs (%** of total Fatty Acids**)	23.08	20.89	11.80	15.95	19.17	16.19
PUFAs (%** of total Fatty Acids**)	56.88	58.59	71.82	60.55	65.15	63.05

[1] nd: not detected.

Table S4. Transition, collision energy, polarity, retention time (RT), calibration curve equation, determination coefficient of each analyte.

Compound	Parent Mass	Product Mass	Collision Energy (eV)	Polarity	RT (min)	Equation	R2
Phenolic acids							
Caffeic acid	180.046	135.902	19	(−)	3.89	$y=2.45545\times10^{-05}+0.807048x$	0.9997
Chlorogenic acid	354.158	191.689	22	(−)	2.36	y=0.00237338+0.0463491x	0.9992
Gallic acid	170.064	126.042	18	(−)	2.05	y=−0.000909657+0.248304x	0.9996
Neochlorogenic acid	354.180	190.875	21	(−)	2.04	y=0.00110944+0.330727x	0.9996
p-Coumaric acid	163.994	119.995	18	(−)	6.16	y=0.00253174+0.386656x	0.9998
Protocatechuic acid	154.055	109.992	18	(−)	2.37	y=0.00114193+0.336255x	0.9996
Sinapic acid	224.081	208.913	16	(−)	6.78	y=0.00292367+0.00171603x	0.9999
Flavonoids							
Apigenin	269.982	118.164	42	(−)	16.98	y=−0.000697875+1.44417x	0.9995
Catechin	290.147	204.072	21	(−)	2.9	y=0.0467805+0.934157x	0.9996
Diosmetin	300.276	284.940	20	(−)	17.67	y=−0.0358838+5.72414x	0.9993
Diosmin	608.363	607.571<br>284.091	13	(−)	8.1	y=−0.000612353+0.15086x	0.9998
Epicatechin	290.159	246.261	18	(−)	3.57	y=−0.116752+4.45105x	0.9995
Epigallocatechin	306.193	125.032	29	(−)	2.47	y=0.000673414+0.0284518x	0.9997
Epigallocatechin gallate	458.179	168.798	21	(−)	3.44	y=−0.0193777+0.909754x	0.9993
Eriodictyol	288.199	151.221	17	(−)	12.67	y=−0.00580681+0.335251x	0.9996
Gallocatechin	306.149	125.017	24	(−)	2.23	$y=6.84895\times10^{-6}+0.223507x$	0.9997
Hesperidin	610.143	301.669	26	(−)	7.78	y=−0.000990985+0.200653x	0.9999
Hesperetin	302.109	286.972	20	(−)	17.37	y=−0.00283134+2.37119x	1.0000
Isoquercetin	464.047	300.702	27	(−)	6.42	$y=-9.13155\times10^{-5}+0.017002x$	0.9996
Isorhamnetin	316.080	300.976	24	(−)	18.08	$y=3.31994\times10^{-5}+0.0163076x$	0.9991
Kaempferol	286.099	240.018	31	(−)	17.46	y=0.00954223+0.0127002x	0.9999
Liquiritigenin	256.166	135.040	18	(−)	12.64	y=0.0106373+0.516127x	0.9997
Liquiritin	418.213	256.024	24	(−)	5.96	y=−0.000116163+0.787945x	0.9994
Luteolin	286.139	132.980	36	(−)	13.74	y=0.000771555+1.10749x	0.9991
Luteolin-4'-O-glucoside	448.270	284.878	21	(−)	8.62	y=0.220464+28.9004x	0.9994
Myricetin	318.121	150.835	26	(−)	9.85	y=−0.00277692+0.21372x	0.9992
Pelargonidin	271.042	121.024	33	(+)	5.20	y=−0.1191133+10.7511356x	0.9995

Compound	Parent Mass	Product Mass	Collision Energy (eV)	Polarity	RT (min)	Equation	R2
Pelargonin	595.241	270.881	33	(+)	2.05	y=0.709165842+229.621287x	0.9998
Procyanidin B1	578.342	407.739	25	(−)	2.3	y=–0.00010714+0.0256914x	0.9995
Procyanidin B2	578.319	408.253	29	(−)	2.83	y=–5.53133×10^{-5}+0.03031x	0.9995
Quercetagetin	318.121	139.101	32	(−)	8.49	y=–0.0184825+1.55459x	0.9993
Quercetagetin-7-O-glucoside	480.253	317.676	25	(−)	3.91	y=–0.0195021+1.5577x	0.9996
Quercetin	302.104	151.049	25	(−)	13.74	y=0.000893219+0.280044x	0.9995
Quercitrin	448.181	300.721	28	(−)	8.3	y=–0.00056989+0.0201204x	0.9995
Rhamnetin	316.116	165.526	25	(−)	20.58	y=–2.61552×10^{-5}+0,0153129x	0.9990
Rutin	610.299	300.561	36	(−)	5.38	y=0.0749463+3.00856x	0.9996
Taxifolin	304.097	285.981	15	(−)	7.19	y=–0.000156342+0.241233x	0.9998
Isoflavonoids							
3'.4'.7-trihydroxyisoflavone	270.133	242.060	24	(−)	9.01	y=–0.00283483+0.618479x	0.9991
4'.6.7-Trihydroxyisoflavone	270.124	240.996	31	(−)	9.66	y=–0.00213213+0.379464x	0.9984
Biochanin A	284.126	268.756	21	(−)	25.08	y=0.000605632+3.02904x	0.9995
Calycosin	284.134	268.921	21	(−)	13.29	y=–0.0561721+5.79216x	0.9998
Calycosin-7-O-D-glycoside	446.305	445.555<br>368.002	14	(+)	10.97	y=–0.000130129+0.0799105x	0.9996
Daidzein	254.287	253.568	15	(−)	12.12	y=–0.000638938+2.11645x	0.9995
Daidzein-7-O-glucuronide	430.263	253.987	32	(−)	4.15	y=0.000207851+0.125476x	0.9991
Daidzin	416.715	254.790	26	(+)	4.02	y=0.000401047+0.0236351x	0.9991
Equol	242.265	121.522	17	(−)	16.38	y=–0.00044859+0.00040563x	0.9991
Formononetin	268.159	252.919	23	(−)	19.69	y=0.00594119+2.83806x	0.9992
Genistein	270.132	133.827	37	(−)	16.66	y=0.0236043+0.993835x	0.9991
Genistein-7-O-glucuronide	446.250	268.842	31	(−)	7.32	y=–0.0350294+0.0875658x	0.9999
Genistin	432.258	268.887	32	(−)	8.43	y=–0.000522399+0.187494x	0.9992
Glycitein	284.179	268.649	24	(−)	13.42	y=–0.000280969+2.77789x	0.9994
Glycitin	446.797	284.768	26	(+)	5.21	y=–4.05352+1140.78x	0.9991
Ononin	430.731	268.785	21	(+)	10.24	y=–0.00378502+0.19401x	0.9999
Puerarin	416.220	295.606	24	(−)	2.79	y=–0.0588534+3.08771x	0.9998
Sissotrin	446.327	283.857	22	(−)	14.06	y=–0.00038752+0.0815812x	0.9996
Sophoricoside	432.293	431.568<br>270.134	19	(−)	8.3	y=0.002776293+1.036527x	0.9997
Chalconoids							
Isoliquiritigenin	256.154	120.019	29	(−)	19.67	y=–0.000802473+1.59052x	0.9996

Compound	Parent Mass	Product Mass	Collision Energy (eV)	Polarity	RT (min)	Equation	R2
Phloretin	274.125	167.912	19	(−)	16.27	y=−0.000383945+1.55783x	0.9997
Phloridzin	436.243	273.890	19	(−)	9.13	y=−0.011024+1.32396x	0.9991
Xanthoxumol	354.240	234.027	21	(−)	31.82	y=−0.00111669+0.00335894x	0.9999
Lignans							
Lariciresinol	360.220	329.991	12	(−)	9.98	y=0.0036846+0.00281203x	0.9999
Matairesinol	358.248	342.987<br>82.914	23	(−)	15.88	y=0.00292574+0.0239047x	0.9992
Secoisolariciresinol	362.276	361.511<br>165.755	11	(−)	9.4	y=0.00211407+0.247818x	0.9988
Coumestan							
Coumestrol	268.134	239.903	26	(−)	17.23	y=0.000117777+0.39815x	0.9995
Phenylethanoil							
Hydroxytyrosol	154.132	124.083	17	(−)	2.25	y=0.000895668+0.106669x	0.9992
Stilbenoids							
Polydatin	390.231	227.693	20	(−)	5.94	y=−0.0120849+4.16746x	0.9991
Reveratrol	228.014	184.140	24	(−)	11.41	y=0.00125676+0.00646586x	0.9992
Internal Stantard	182.748	136.996	26	(−)	15.68		

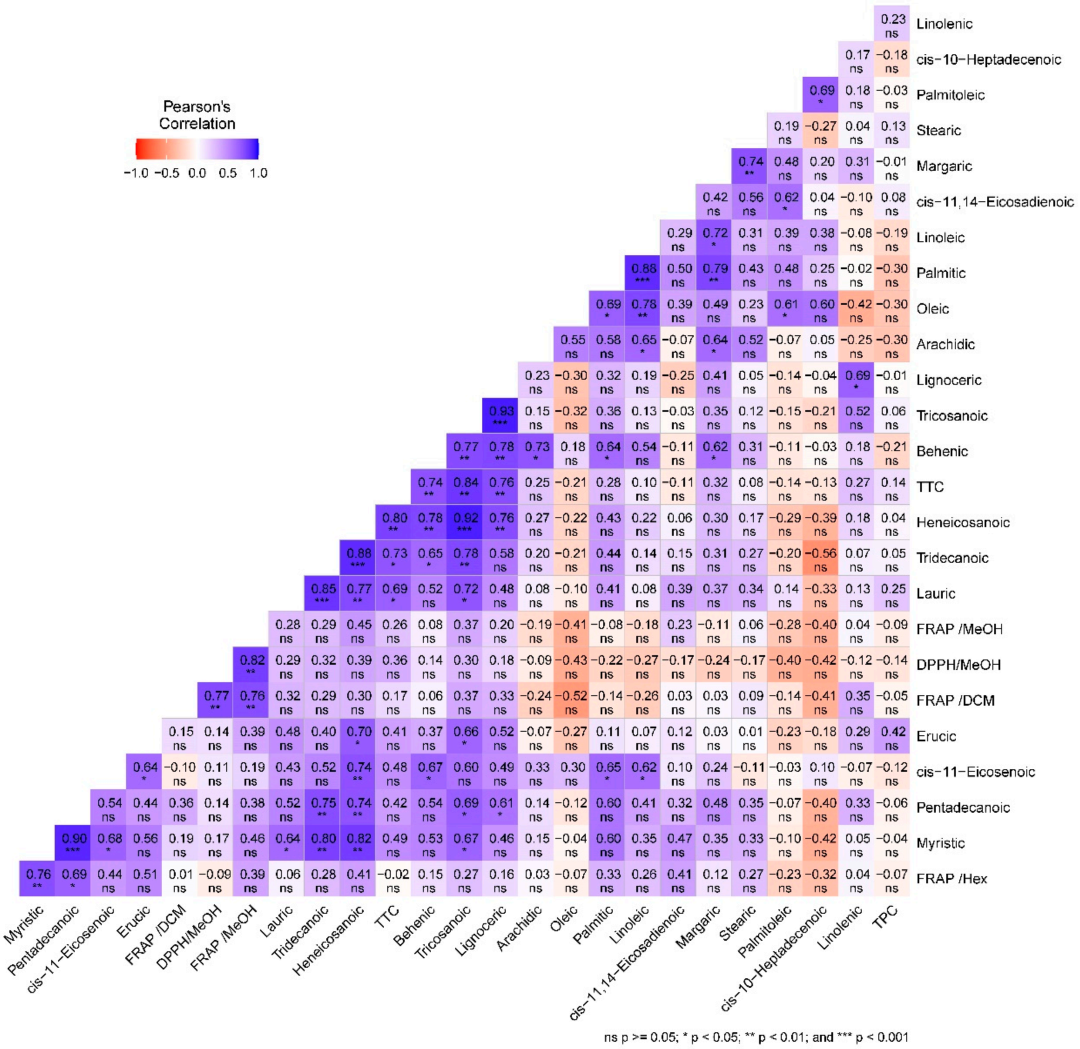

Figure S1: Matrix of the Pearson correlation coefficients.

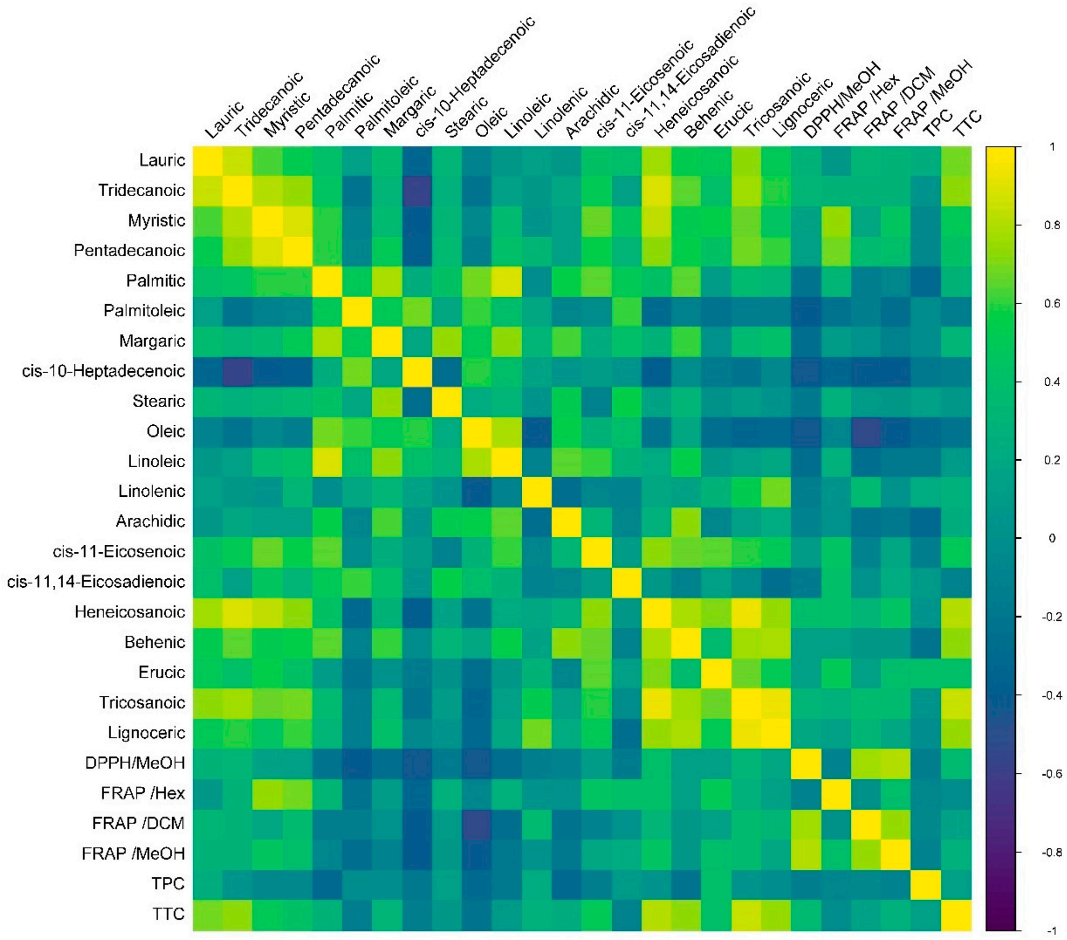

Figure S2 Correlation heatmap depicting the relationships between phytochemical components and antioxidant properties.
